# The effectiveness of mindfulness-based cognitive therapy combined with medication therapy in preventing recurrence of major depressive disorder in convalescent patients

**DOI:** 10.3389/fpsyg.2022.882006

**Published:** 2022-08-18

**Authors:** Hui-Rong Guo, Jun-Ru Wang, Ya-Li Wang, Bai-Ling Huang, Xu-Huan Yang, Yu-Ming Ren

**Affiliations:** ^1^Department of Psychiatry, The First Affiliated Hospital of Zhengzhou University, Zhengzhou, China; ^2^Department of Psychiatry, People's Hospital of Zhengzhou, Zhengzhou, China; ^3^Office of Academic Studies, Xinxiang Medical University, Xinxiang, China

**Keywords:** mindfulness-based cognitive therapy, prevention, convalescent depression, recurrence, MDD

## Abstract

**Objective:**

This study aims to investigate the effectiveness of mindfulness-based cognitive therapy (MBCT) combined with medication therapy in preventing the recurrence of major depressive disorder (MDD) in convalescent patients.

**Methods:**

A total of 130 patients with convalescent MDD were enrolled in this prospective study. Sixty-five patients were assigned to the experimental group and received medication therapy combined with MBCT, and 65 patients were assigned to the control group and treated with medication alone. The recurrence rate and related hormonal changes were compared between the two groups.

**Results:**

After 1 year of MBCT intervention, eight patients experienced recurrence in the experimental group, a recurrence rate of 12.31%, and 19 patients experienced recurrence in the control group, a recurrence rate of 29.23%. The Hamilton Depression Rating Scale (HAM-D) and the World Health Organization Quality of Life Scale (WHOQOL-BREF) scores in both the experimental and the control groups were significantly improved after treatment (*P* < 0.05). The difference in the HAM-D scores before and after treatment in the experimental group was 16.74 ± 4.54; this was significantly higher than that of the control group (8 ± 3.89, *P* < 0.0001). The WHOQOL-BREF scores in the experimental group were significantly improved compared with those of the control group (*P* < 0.0001). The differences in the levels of corticotrophin-releasing hormone (CRH), adrenocorticotropic hormone, and cortisol before and after treatment in the experimental group and the control group were statistically significant (*P* < 0.05). The difference in CRH before and after treatment in the experimental group was 16.8 ± 7.2, which was higher than that of the control group (2.75 ± 9.27, *P* < 0.0001). The intervention with MBCT had a significant impact on the recurrence of MDD [β = 1.206, *P* = 0.039, 95% (confidence interval) CI = 0.0790–1.229]. The difference in the HAM-D scores also had a significant impact on the recurrence of MDD (β = 1.121, *P* = 0.0014, 95% CI = 0.805–0.976).

**Conclusion:**

Compared with medication therapy alone, the use of MBCT combined with medication therapy can effectively prevent the recurrence of MDD in convalescent patients.

## Introduction

Major depressive disorder (MDD) is characterized by depression, slow thinking, cognitive dysfunction, decreased volitional activity, and negative suicide ideation. The prevalence and recurrence rate of MDD are high, seriously affecting the quality of life and social functioning of patients. The incidence of MDD is influenced by many factors, including life events, genetic factors, biological factors, cognitive factors, and personality factors. The goal of treatment in the acute stage is to control symptoms, usually with medication therapy. However, after treatment with selective serotonin reuptake inhibitors (SSRIs) to control depressive symptoms, many patients still do not maintain a stable psychological state for a sustained period of time during the rehabilitation period and are highly likely to have a recurring depressive episode. The recurrence rate within 1 year is 33%, and 80% of patients experience greater than two recurrences (Xiao et al., 2017).

Mindfulness-based cognitive therapy (MBCT) is a psychotherapy method developed by combining mindfulness-based stress reduction and cognitive therapy that can effectively prevent the recurrence of MDD. This therapy emphasizes the patient accepting their true feelings in the present moment in a natural state without deliberately changing their existing ideas, thus improving self-acceptance. The practice of MBCT places emphasis on perception of the details of daily life and encourages patients to improve their sense of well-being through their perception of daily activities. At present, MBCT is widely used in many fields, such as clinical medicine, clinical psychology, sports, military training, and education, and has achieved remarkable results (Rofiq Muhammad et al., 2021). In previous studies, MBCT was often used as an adjuvant treatment for patients with improved symptoms of post-acute depression. Some studies reported the effectiveness of MBCT combined with drug therapy vs. drug alone in the control of MDD, but there was no agreement, and the changes of hypothalamic-pituitary-adrenal axis related hormone levels were not simultaneously detected (SangHyuk and Joon, 2021).

This study aims to investigate the effectiveness of MBCT combined with medication therapy in preventing the recurrence of convalescent MDD patients, and further to provide theoretical basis for the application of MBCT.

## Data and methods

### Clinical data

From January 2017 to June 2019, 130 patients with convalescent depression treated in the psychiatric department of our hospital were included in this prospective study. The experimental group consisted of 65 patients received MBCT combined with medication therapy. The control group included 65 patients receiving medication therapy alone. This study protocol was formulated in accordance with the requirements of the Declaration of Helsinki of the World Medical Association. It was approved by the Scientific Research and Clinical Trial Ethics Committee of the First Affiliated Hospital of Zhengzhou University (NO: 2019-KY-0508-003), and the informed consent forms were obtained from all patients.

### Inclusion and exclusion criteria

Inclusion criteria: 1. Diagnosed of GDM according to the diagnostic criteria of MDD in the International Classification of Diseases 10^th^ Revision (People's Medical Publishing House., 1993); 2. Age ranged from 18–60 years old; 3. Patients were in the convalescent phase of MDD and their symptoms were improved; 4. The Hamilton Depression Rating Scale (HAM-D_24_) score was ≤7 points; 5. Patients had a lucid mental state and could cooperate with MBCT.

Exclusion criteria: 1. Patients with a previous history of manic episode and schizophrenia; 2. Patients with history of multiple organ dysfunction; 3. Two Patients with history of malignant tumor; 4. Pregnant patients.

### Medication therapy

All patients in this study were in the maintenance and rehabilitation period of MDD and the patients in both groups took antidepressant medication regularly in the acute phase. Priority was given to the SSRI fluoxetine (20–40 mg/d). If the patient was experiencing obvious agitation, a sedative such as mirtazapine (15–30 mg/d) was given. If the patient developed obsessive symptoms, dose of Fluoxetine was increased (to 60 mg/d). Fluvoxamine (100–200 mg/d) or sertraline (50–150 mg/d) in combination with aripiprazole (5–10 mg/d) should be given if patients developed psychotic symptoms.

### Mindfulness-based cognitive therapy

Patients in the experimental group received MBCT in addition to regular medication. The MBCT treatment process for all patients was led by two regular psychotherapists who had been trained in MBCT for more than 2 years, one principal leader and one co-leader, to ensure good quality of MBCT intervention. Before treatment, psychotherapists evaluated the patient's condition and basic situation, communicated with the patient about treatment methods and processes, and established a treatment alliance. The patient signed the informed consent form and relevant information was issued to ensure the patient fully understood the plan of care. The period of centralized intervention was 6 months, with 13 people in a group. Treatment was conducted once a week in assigned groups. Each treatment lasted for 2.5 h, for a total of 24 treatments. After weekly group treatments were completed, the daily practice of MBCT continued for 6 months, supervised through a WeChat group. Specific methods were as follows. (1) Whole body scan: instruct the patient to scan all parts of their body from bottom to top, paying attention to bodily sensations, thoughts, and emotions, accepting them, and feeling the present moment. (2) Mindfulness breathing: instruct the patient to consciously pay attention to the changes in their body with breathing, especially the movement of their abdomen, concentrating their attention and perceiving other physical feelings, emotions, and thoughts at the same time. (3) Mindfulness raisin-eating exercise: opening of the five senses: sight, touch, smell, taste, and hearing. Patients slowly eat a raisin and note their body's sensations, thoughts, and emotions. (4) Mindfulness walking: Patients stand quietly, relaxing their bodies, walking slowly, focusing on the feeling of their feet leaving and contacting the ground, and perceive their body's experience of movement. (5) Three minutes of breathing space: patients sit with their eyes closed, focusing on the present experience of breathing, and expand this experience into their life. After each exercise, group members were invited to share their experiences. A WeChat group was established, through which daily homework was assigned, audio for exercise guidance was distributed, and the completion of individual mindfulness exercises was supervised. Patients were required to complete 1 h of mindfulness training outside the group each day. The total mindfulness training time of each patient after enrollment was counted.

### Observation index and comparison method

(1) Comparison of clinical effectiveness: The HAM-D scores before and after intervention were counted, and the scores of the two groups after 6 months were compared. The quality of life of the patients in the two groups was evaluated using the World Health Organization Quality of Life Scale (WHOQOL-BREF) scale before and after treatment. (2) Venous blood was drawn on an empty stomach after approximately 6 months of treatment, and the levels of hypothalamic–pituitary–adrenal (HPA) axis related hormones, including adrenocorticotropic hormone (ACTH), corticotropin-releasing hormone (CRH), and corticosterone (CORT), were measured. (3) According to the HAM-D score and the diagnostic criteria for MDD, the recurrence rate of MDD in the two groups was evaluated. The correlation of MBCT with the clinical effectiveness and hormone levels in patients with MDD in the convalescent period was analyzed, and its influence on the recurrence of MDD was calculated. In the regression analysis, when the score difference between the HAM-D and the WHOQOL-BREF was <10, it was assigned as one, and when the score difference was higher than 10, it was assigned as zero. When the hormone level after treatment subtracted from the hormone level before treatment yielded a positive value, it was assigned as zero, and when it yielded a negative value, it was assigned as one. In the case of a recurrence of MDD it was assigned as one, otherwise it was assigned as zero.

### Data statistical analysis

The data were statistically analyzed using R package version 3.51. Measurement data were evaluated for normality and homogeneity of variance. Normally distributed measurement data were expressed as the mean ± standard deviation ($\overset{\bar{}}{x}$ ± SD) and compared using an independent sample *t*-test; non-normally distributed measurement data were described as interquartile and compared between the two groups using a rank sum test. Count data were compared using the *X*^2^ test. Binary data were analyzed using a logistic regression analysis. *P* < 0.05 was considered to be statistically significant.

## Results

### Comparison of the baseline data between the two groups

The baseline data of the patients between the two groups were compared. There were no significant differences in age, gender, marital status, education level, occupation, and duration of disease between the two groups. Both groups had the highest proportion of patients with college education and above, and the highest proportion of mental workers. The disease course of most patients was longer than 1 year (Table 1).

**Table 1:** Comparison of baseline data between the two groups.

**Variable**	**Experimental group**	**Control group**	**Statistical value (t/χ)**	***P*-value**
***n***	65	65		
**Age**	43.09 ± 12.26	39.92 ± 11.35	1.126	0.291
**Gender**
Male	32	31	0.031	0.5
Female	33	34		
**Marriage state**
Married	48	46	1.056	0.59
Unmarried	9	13		
Divorced	8	6		
**Education level**
Middle school	13	16	1.041	0.792
Junior college	11	9		
Bachelor	37	34		
Graduate	4	6		
**Occupational type**
Physical	19	21	0.143	0.425
Mental	46	44		
**Course of disease (years)**
<1	11	8	1.25	0.534
1~3	27	33		
>3	27	24		

### Comparison of the clinical indexes before and after intervention between the two groups of patients

After a year of MBCT, psychological assessments of the patients were conducted. Eight patients in the experimental group experienced recurrence, with a recurrence rate of 12.31%. Nineteen patients in the control group experienced recurrence, with a recurrence rate of 29.23%, revealing that the recurrence rate was significantly lower in the experimental group than in the control group (*P* < 0.001). The HAM-D scores in both the experimental and the control groups were significantly improved after treatment. A paired *t*-test yielded *P* < 0.05, and the results of the WHOQOL-BREF scores were similar. The difference between the HAM-D score before and after treatment in the experimental group was 16.74 ± 4.54, which was significantly higher than that of the control group (8 ± 3.89, *P* < 0.0001); the WHOQOL-BREF scores in the experimental group were significantly improved compared with the scores of the control group (*P* < 0.0001, Table 2).

**Table 2:** Comparison of clinical data between the two groups after acute phase treatment and MBCT.

**Variable**	**Experimental group**		**Control group**		**Statistical value (t/χ)**	***P*-value**
	**Before treatment**	**After treatment**	**Before treatment**	**After treatment**		
HAMD score	28.17 ± 4.04*	11.43 ± 2.08*	30.26 ± 1.11^#^	22.26 ± 3.77^#^		
HAMD difference	16.74 ± 4.54		8 ± 3.89		11.787	<0.001
WHOQOL score	32.49 ± 3.74*	77.68 ± 6.37*	33.54 ± 3.56^#^	31.94 ± 2.7^#^	2.933	
WHOQO difference	45.18 ± 7.68		1.6 ± 4.4		42.634	<0.001
Recurrence rate	8(12.31%)		19(29.23%)		17.591	<0.001

* For the experimental group, paired sample t-test is performed before and after MBCT, P < 0.05.

# For the control group, paired sample t-test is performed before and after MBCT, P < 0.05.

### Comparison of the hormone levels between the two groups before and after intervention

The hormone indexes of the two groups were measured before and after treatment. The differences in the levels of CRH, ACTH, and CORT before and after treatment in the experimental and the control groups were statistically significant (*P* < 0.05). The difference in CRH before and after treatment in the experimental group was 16.8 ± 7.2, which was higher than in the control group (2.75 ± 9.27, *P* < 0.0001). The difference in the ACTH scores before and after treatment in the experimental group was 25.11 ± 6.98, which was significantly higher than in the control group (14.31 ± 7.12, *P* < 0.0001). There was also significant statistical difference in CORT before and after treatment (*P* < 0.001) (Table 3).

**Table 3:** Comparison of hormone levels between the two groups before and after treatment.

**Variable**	**Experimental group**		**Control group**		**Statistical value (t/χ)**	***P*-value**
	**Before treatment**	**After treatment**	**Before treatment**	**After treatment**		
CRH	32.66 ± 6.74*	15.86 ± 2.97*	32.28 ± 6.38^#^	29.52 ± 5.29^#^		
CRH difference	16.8 ± 7.2		2.75 ± 9.27		9.65	<0.001
ATCH	53.51 ± 7.07*	28.4 ± 1.66*	52.78 ± 6.81^#^	38.48 ± 1.64^#^		
ATCH difference	25.11 ± 6.98		14.31 ± 7.12		8.728	<0.001
CORT	120.69 ± 7.52*	51.4 ± 9.4*	119.88 ± 7.66^#^	122.86 ± 8.22^#^	
CORT difference	69.29 ± 11.49		2.98 ± 11.95		35.153	<0.001

* For the experimental group, paired sample t-test is performed before and after MBCT, P < 0.05.

# For the control group, paired sample t-test is performed before and after MBCT, P < 0.05.

### Logistic regression analysis of related factors affecting recurrence in the two groups

Clinical factors, such as a difference in the HAM-D score and hormone level changes, were substituted into the regression model. Intervention with MBCT had a significant impact on the recurrence of MDD [β = 1.206, *P* = 0.039, 95% confidence interval (CI) = 0.0790–1.229]. The difference in the HAM-D score also had a significant impact on the recurrence of MDD (β = 1.121, *P* = 0.0014, 95% CI = 0.805–0.976). CORT had a certain influence, but the degree of influence was low (*P* = 0.043, β = 1.143). No significant effect was observed from the other factors (Table 4).

**Table 4:** Analysis of related influencing factors of depression recurrence.

**Variable**	**β value**	**Wals value**	***P*-value**	**Exp (β)**	**95%CI**
HAMD difference	1.121	6.052	0.014	0.886	0.805–0.976
WH difference	0.307	0.255	0.614	1.359	0.413–4.484
CRH difference	0.693	1.689	0.193	1.999	0.703–1.229
ATCH difference	0.133	0.065	0.799	0.875	0.314–2.441
COAT difference	1.143	0.606	0.043	0.886	0.979–1.056
MBCT intervention	1.206	2.799	0.039	0.299	0.0790–1.229

## Discussion

With the continuous development of social economy and an ever-quickening pace of life, the incidence of MDD is increasing (Lu et al., 2017). The prevalence of MDD is higher than that of diabetes, hypertension, and other traditional chronic diseases. At present, it is the second most common disease in the world. Epidemiological studies have revealed that at present, the lifetime prevalence of depression in China is about 3.4%. Based on this, it is estimated that about 44 million people suffer from the disease (Yuan, 2020). The burden of MDD seriously theratens the stability of patients' families and society. The goal of treating acute depression is “symptom cure,” and first drug treatment is effective. The recurrence rate of patients in the rehabilitation stage of MDD is very high, and the treatment effects and prognosis after multiple recurrences are poor (Buckman et al., 2018; Shallcross et al., 2018).

The practice of MBCT is an exercise in perception, helping patients to adopt specific orientations or attitudes and learn to respond to challenges in life with a new way of responding to challenges rather than avoiding them. The patients are trained to focus on their current experiences and feelings, such as curiosity, openness, and acceptance, to cultivate a gentle, nonjudgmental, and receptive attitude, to be friendly to and care for themselves, and thereby break the cycle of negative cognition in the psychological pathogenesis of depression (Gu et al., 2015; Wang et al., 2021). The preivous study showed that MBCT could regulate the existed impaired neuroplasticity in patients with depression (Yu et al., 2020).

In this study, there were no significant statistical differences at a baseline level between the two groups, suggesting that the research sample avoided a sampling error to a certain extent. After the additional MBCT intervention, including 6 months of group treatment and 6 months of daily treatment, there was a significant difference in recurrence rates between the study group and the control group. In Addition, the differences in the values of the HAM-D and WHOQOL-BREF scores in the experimental group also improved significantly, suggesting that MBCT combined with medication therapy is better than medication therapy alone in improving depressive symptoms and psychosocial function. Although there is a standardized model for MBCT treatment, results may vary in part from study to study due to different understandings and proficiency among MBCT operators. Results of a randomized controlled study showed that MBCT therapy alone and antidepressant maintenance therapy alone were associated with low recurrence rates, effective symptom relief, and improved quality of life (Kuyken et al., 2015). The addition of MBCT was associated with lower 1- and 2-year recurrence rates (odds ratio 0.45) in a 2-year follow-up study (Meadows et al., 2014).

The relationship between the occurrence of depression and hormone-related factors in serum and cerebrospinal fluid has attracted increased attention (Bangsgaard and Ottesen, 2017). A previous study (Mikulska et al., 2021) revealed that the long-term overactivation of the HPA axis can lead to abnormalities in human psychological and physiological function. Because CRH is at the top of the HPA axis and is at the center of the stress response, the abnormal expression of CRH is closely related to the expression of amino acid neuropeptides related to mental diseases. A relevant animal experiment has also confirmed that blocking the function of the HPA axis can affect the function of the CRH1 antagonistic receptor (Keller et al., 2017). Compared with the general population, the expression of CRH increases in patients with severe depression (van der Doelen et al., 2014). It demonstrated that ACTH and CORT lead to increased secretion of adrenal glucocorticoid and cortisol; elevated levels of ACTH expression directly lead to hyperfunction of the HPA axis, affecting the normal rhythm of physiology and psychology and affecting the cognitive function of the brain (Karling et al., 2016). In this study, the levels of HPA-related hormones in the experimental and the control groups were compared before and after treatment. After 6 months of intervention, the differences in the levels of CRH, ACTH, and CORT before and after treatment in the experimental and the control groups were statistically significant; the difference in CRH and ACTH in the experimental group was higher than in the control group. Previous studies have reached similar conclusions. Results of a systematic review show that targeting hypothalamic-pituitary-adrenal axis hormones and sex steroids can improve cognition in patients with mood disorders and schizophrenia, suggesting that HPA axis related hormones may play a role in the development of depression (Soria et al., 2018).

The results of this present study revealed that the possible mechanism of MBCT to improve depressive symptoms and reduce the recurrence rate is to intervene in the regulatory function of the HPA axis. However, the related mechanism still needs to be verified by systematic *in vivo* and *in vitro* experiments. In recent years, a previous study (Wong et al., 2018) revealed that mindfulness meditation training can significantly control and improve the sexual hormone levels of perimenopausal women. Mindfulness training emphasizes focused and concentrated breathing: deep, long, and slow breathing, causing the sympathetic nervous system to relax, effectively correcting the dysfunction of the autonomic nervous system (Li et al., 2015).

The results of a related previous study (Vittengl et al., 2016) revealed that the recovery of the social function of depression was significantly correlated with age, gender, treatment methods, social factors, methods of treatment, depressive symptoms, psychosocial function, and hormone changes. Before and after treatment results were substituted into the prediction model for analysis, where it was revealed that MBCT intervention and difference in the HAM-D scores had a significant impact on the recurrence of depression. And the difference in the CORT had a certain influence, but the degree of influence was low, which may be due to the short follow-up period of this study. Therefore, MBCT can effectively prevent the recurrence of MDD in patients in the convalescent phase, and a change in the HAM-D scores can effectively reflect the risk of recurrence of MDD.

A shortcoming of this study was that the follow-up was shorter, just 1 year, which may weaken the generalizability of the results. Another limitation is that only serological indicators of brain function were collected and analyzed in this study, and imaging indicators were not included. In the follow-up study, we will extend the follow-up period of enrolled patients, expand the sample size to obtain long-term efficacy results, and include more potential research indicators to provide a further theoretical basis for the follow-up study.

## Conclusion

In summary, compared with medication therapy alone, MBCT combined with medication therapy can effectively prevent the recurrence of MDD in patients in the convalescent phase and is worthy of wide clinical implementation.

## Data availability statement

The original contributions presented in the study are included in the article/supplementary material, further inquiries can be directed to the corresponding authors.

## Ethics statement

The studies involving human participants were reviewed and approved by Ethics Committee of The First Affiliated Hospital of Zhengzhou University. The patients/participants provided their written informed consent to participate in this study.

## Author contributions

Conception and design of the research: H-RG and Y-MR. Acquisition of data: J-RW and X-HY. Analysis and interpretation of the data: B-LH. Statistical analysis: Y-LW and J-RW. Obtaining financing: H-RG. Writing of the manuscript, critical revision of the manuscript for intellectual content, and read and approved the final draft: all authors. All authors contributed to the article and approved the submitted version.

## Funding

This work was supported by Science and Technology Talents Overseas Training project, Health Commission Henan Province (2018014).

## Conflict of interest

The authors declare that the research was conducted in the absence of any commercial or financial relationships that could be construed as a potential conflict of interest.

## Publisher's note

All claims expressed in this article are solely those of the authors and do not necessarily represent those of their affiliated organizations, or those of the publisher, the editors and the reviewers. Any product that may be evaluated in this article, or claim that may be made by its manufacturer, is not guaranteed or endorsed by the publisher.

## References

[B1] Bangsgaard E. O. Ottesen J. T. (2017). Patient specific modeling of the HPA axis related to clinical diagnosis of depression. Bellman Prize in Mathematical Biosciences. 287, 24–35. 10.1016/j.mbs.2016.10.007 27816534

[B2] Buckman J. E. Underwood A. Clarke K. Saunders R. Hollon S. D. Fearon P.. (2018). Risk factors for recurrence and recurrence of depression in adults and how they operate: a four-phase systematic review and meta-synthesis. Clin. Psychol. Rev. 64, 13–38. 10.1016/j.cpr.2018.07.005 30075313 PMC6237833

[B3] Gu J. Strauss C. Bond R. Cavanagh K. (2015). How do mindfulness-based cognitive therapy and mindfulness-based stress reduction improve mental health and wellbeing? A systematic review and meta-analysis of mediation studies. Clin. Psychol. Rev. 37, 1–12. 10.1016/j.cpr.2015.01.006 25689576

[B4] Karling P. Wikgren M. Adolfsson R. Norrback K. F. (2016). Hypothalamus-Pituitary-Adrenal Axis Hypersuppression Is Associated with Gastrointestinal Symptoms in Major Depression. J. Neurogastroenterol. Motil. 22, 292–303. 10.5056/jnm15064 26507800 PMC4819868

[B5] Keller J. Gomez R. G. Williams G. H. Lembke A. Lazzeroni L. Murphy G. M. Jr. (2017). HPA axis in major depression: cortisol, clinical symptomatology and genetic variation predict cognition. Mol. Psychiatry. 22, 527–536. 10.1038/mp.2016.120 27528460 PMC5313380

[B6] Kuyken W. Hayes R. Barrett B. Byng R. Dalgleish T. Kessler D.. (2015). Effectiveness and cost-effectiveness of mindfulness-based cognitive therapy compared with maintenance antidepressant treatment in the prevention of depressive relapse or recurrence (PREVENT): a randomised controlled trial. Lancet. 386, 63–73. 10.1016/S0140-6736(14)62222-4 25907157

[B7] Li D. H. Qian A. Y. Wang T. Yang Q. L. Su X. J. (2015). Effect of meditation training with health education on the clinical effect of low-dose hormone replacement therapy for perimenopausal syndrome. He Bei Yi Yao. 37, 2474–2476. 10.3969/j.issn.1002-7386.2015.16.024

[B8] Lu J. Li L. J. Xu X. F. (2017). Chinese Guidelines for the Prevention and Treatment of Depressive Disorders (Second Edition) Interpretation: Assessment and Diagnosis. Zhong Hua Jing Shen Ke Za Zhi. 50, 169–171. 10.3760/cma.j.issn.1006-7884.2017.03.003

[B9] Meadows G. N. Shawyer F. Enticott J. C. Graham A. L. Judd F. Martin P. R.. (2014). Mindfulness-based cognitive therapy for recurrent depression: A translational research study with 2-year follow-up. Aust. N. Z. J. Psychiatry. 48, 743–755. 10.1177/0004867414525841 24595511

[B10] Mikulska J. Juszczyk G. Gawrońska-Grzywacz M. Herbet M. (2021). HPA axis in the pathomechanism of depression and schizophrenia: new therapeutic strategies based on its participation. Brain Sci. 11, 1298. 10.3390/brainsci11101298 34679364 PMC8533829

[B11] People's Medical Publishing House. (1993). ICD-10 Classification of Mental and Behavioral Disorders (10th edition), 54–57.

[B12] Rofiq Muhammad R. Gian Sugiana S. Feida Noorlaila I. (2021). Effect of mindfulness-based cognitive therapy on academic grit among university student. Curr. Psychol. 40, 1–10. 10.1007./S12144-021-01795-4

[B13] SangHyuk L. Joon C. S. (2021). Cognitive behavioral therapy and mindfulness-based cognitive therapy for depressive disorders. Adv. Exp. Med. Biol. 1305, 295–310. 10.1007/978-981-33-6044-0_16 33834406

[B14] Shallcross A. J. Willroth E. C. Fisher A. J. Dimidjian S. Gross J. J. Visvanathan P. D.. (2018). Recurrence/recurrence prevention in major depressive disorder: 26-month follow-up of mindfulness-based cognitive therapy versus an active control. Behav. Ther. 49, 836–849. 10.1016/j.beth.2018.02.001 30146148 PMC6112178

[B15] Soria V. González-Rodríguez A. Huerta-Ramos E. Usall J. Cobo J. Bioque M.. (2018). Targeting hypothalamic-pituitary-adrenal axis hormones and sex steroids for improving cognition in major mood disorders and schizophrenia: a systematic review and narrative synthesis. Psychoneuroendocrinology. 93, 8–19. 10.1016/j.psyneuen.2018.04.012 29680774

[B16] van der Doelen R. H. Deschamps W. D'Annibale C.. (2014). Early life adversity and serotonin transporter gene variation interact at the level of the adrenal gland to affect the adult hypothalamo-pituitary-adrenal axis. Transl. Psychiatry. 4, e409. 10.1038/tp.2014.57 25004389 PMC4119224

[B17] Vittengl J. R. Clark L. A. Thase M. E. Jarrett R. B. (2016). Longitudinal social-interpersonal functioning among higher-risk responders to acute-phase cognitive therapy for recurrent major depressive disorder. J. Affect Disord. 199, 148–156. 10.1016/j.jad.2016.04.017 27104803 PMC4862892

[B18] Wang Y. Fu C. Liu Y. Li D. Wang C. Sun R.. (2021). A study on the effects of mindfulness-based cognitive therapy and loving-kindness mediation on depression, rumination, mindfulness level and quality of life in depressed patients. Am J. Transl. Res. 13, 4666–4675. 34150046 PMC8205847

[B19] Wong C. Yip B. H. Gao T.. (2018). Mindfulness-based stress reduction (MBSR) or psychoeducation for the reduction of menopausal symptoms: a randomized, controlled clinical trial. Sci. Rep. 8, 6609. 10.1038/s41598-018-24945-4 PMC5919973 29700350

[B20] Xiao L. Feng L. Zhu X. Q.. (2017). A national survey of residual symptoms in Chinese depressive patients after acute phase treatment. Zhong Hua Jing Shen Ke Za Zhi 50, 175–181. 10.3760/cma.j.issn.1006-7884.2017.03.005

[B21] Yu C. Li A. Li X. Chen Z. Wang P. Daskalakis D. Z.. (2020). Impaired LTD-like motor cortical plasticity in female patients with major depression disorder. Neuropharmacology. 179, 108268. 10.1016/j.neuropharm.2020.108268 32791084

[B22] Yuan Y. G. (2020). Artisanal scholarship-anticipating suicidal behavior in depressed patients! *Zhong Guo Quan Ke Yi Xue*. 23, 3245.

